# p53-Induced LINC00893 Regulates RBFOX2 Stability to Suppress Gastric Cancer Progression

**DOI:** 10.3389/fcell.2021.796451

**Published:** 2022-01-19

**Authors:** Xinde Ou, Xingyu Zhou, Jin Li, Jinning Ye, Haohan Liu, Deliang Fang, Qinbo Cai, Shirong Cai, Yulong He, Jianbo Xu

**Affiliations:** ^1^ Department of Gastrointestinal Surgery, The First Affiliated Hospital, Sun Yat-sen University, Guangzhou, China; ^2^ Laboratory of General Surgery, The First Affiliated Hospital, Sun Yat-sen University, Guangzhou, China; ^3^ Digestive Disease Center, The Seventh Affiliated Hospital, Sun Yat-sen University, Shenzhen, China

**Keywords:** p53, LINC00893, gastric cancer, RBFOX2, epithelial-mesenchymal transition

## Abstract

Long noncoding RNAs (lncRNAs) have been reported to regulate diverse tumorigenic processes. However, little is known about long intergenic non-protein coding RNA 00893 (LINC00893) and its role in gastric cancer (GC). Herein we investigated its biological functions and molecular mechanism in GC. LINC00893 was decreased in GC tissues but significantly elevated in AGS cells after treatment with Nutlin-3. In GC patients, it was found that low expression of LINC00893 was correlated with tumor growth, metastasis and poor survival. Functionally, overexpression of LINC00893 suppressed the proliferation, migration and invasion of GC cells. Mechanistically, LINC00893 regulated the expression of epithelial-mesenchymal transition (EMT)-related proteins by binding to RNA binding fox-1 homolog 2 (RBFOX2) and promoting its ubiquitin-mediated degradation, thus suppressing the EMT and related functions of GC. In addition, the transcription factor p53 can regulate the expression of LINC00893 in an indirect way. Taken together, these results suggested that LINC00893 regulated by p53 repressed GC proliferation, migration and invasion by functioning as a binding site for RBFOX2 to regulate its stability and the expression of EMT-related proteins. LINC00893 acts as a tumor-inhibiting lncRNA that is induced by p53 in GC and regulates EMT by binding to RBFOX2, thus providing a novel experimental basis for the clinical treatment of GC.

## Introduction

Gastric cancer (GC) is one of the leading causes of cancer-related human mortalities, and remains the fourth leading cause of cancer-related deaths in 2020 (Sung et al., 2021). Strikingly, GC cases in China account for about 42% of all cases worldwide, mainly owing to the high prevalence of *Helicobacter pylori* infection (Cai et al., 2021). Moreover, although massive efforts have been made by researchers to develop noninvasive biomarkers and therapeutic targets to reduce GC mortality, little success has been achieved in increasing the overall survival rate. Nonresectable or metastatic GC is always associated with poor prognosis, and patients can get minimal benefit from systemic chemotherapeutic approaches (Mehta et al., 2020). Therefore, novel biomarkers for improving the early diagnosis, tumor grading and prognostic evaluation of GC are urgently needed.

Owing to the rapid development in high-throughput sequencing, it has been determined that nonprotein coding RNA accounts for the vast majority of RNA transcribed from human genome, which indicates that a relatively small number of effectors is dedicated to be regulated by a large group of RNA regulators (Katayama et al., 2005; Djebali et al., 2012). Among these newly discovered nonprotein RNA elements, long noncoding RNAs (lncRNAs) have been widely reported to function as key regulators in multiple cellular processes, such as cell differentiation, development, and disease pathogenesis (Kung et al., 2013). To date, the expression profile of lncRNAs in GC have been screened by different groups and several disordered lncRNAs related to GC carcinogenesis have been investigated, such as MALAT1, HOTAIR, and UCA1(Liu et al., 2014; Li et al., 2017; Wang et al., 2019). LncRNAs are critical regulators of GC initiation, progression, dissemination and immune evasion (Dragomir et al., 2020). It has also been widely proved that there is a close connection between lncRNAs and gastric cancer metastasis. Some of them are reported to promote the metastasis of gastric cancer, such as lncRNA UBE2CP3, HOTAIR and GMAN (Wei et al., 2020; Xu et al., 2020; Li et al., 2021). Meanwhile, lncRNAs such as lnc-CTSLP4 and lnc-LEMGC can inhibit the metastasis of gastric cancer through different ways (Pan et al., 2021; Zhang et al., 2022).

The gene *TP53*, encoding p53 protein, is the most frequently mutated tumor suppressor gene in diverse human cancers and, therefore, one of the most broadly investigated regulatory networks (Vousden and Prives, 2009; Allen et al., 2014). For decades, when studying the regulatory mechanisms and downstream targets which execute the biological functions of p53, researchers mainly focused on the products of protein-coding genes (Donehower et al., 2019). Recently, it was discovered that lncRNAs were key regulatory players which could shape p53 activity and biological outcomes. The p53 regulatory networks are now reported to be intervened by multitudinous p53-regulated lncRNAs either directly or indirectly in cancers (Jain, 2020). LincRNA-p21, GUARDIN, NEAT1 and TP53TG1 are transcriptionally regulated by p53 (Huarte et al., 2010; Diaz-Lagares et al., 2016; Mello et al., 2017; Hu et al., 2018), while DDSR1 and LINP1 are induced by p53 in indirect ways (Sharma et al., 2015; Zhang et al., 2016).

In this study, with bioinformatic and statistical analysis of high-throughput sequencing data, we found that the expression of LINC00893 was significantly reduced in GC tissues but increased in AGS cells after increasing p53, so we further investigated LINC00893 in GC. We discovered that LINC00893 was regulated by p53 in an indirect way, while low expression of LINC00893 was positively associated with GC growth, metastasis and indicated a poor survival of GC patients. We further revealed that LINC00893 inhibited proliferation, metastasis and EMT processes of GC cells through binding to the protein RBFOX2 and promoting its degradation. Our new findings on the p53/LINC00893/RBFOX2 regulatory axis illustrate its potential as a target for GC diagnostic and therapeutic development.

## Materials and Methods

### Cell Culture and Cell Transfection

The GC cell lines AGS, MKN28 and MKN45 were obtained from the Type Culture Collection of the Chinese Academy of Sciences (China). The normal gastric epithelial cell line GES-1 was purchased from the Lab Animal Center of the Fourth Military Medical University (China). All cell lines were routinely tested and had negative results for *mycoplasma*. All cells except AGS were maintained in DMEM (Invitrogen) and AGS in F12 (Invitrogen) supplemented with 10% fetal bovine serum (GIBCO) in an incubator with 5% CO2 and 95% room air at 37°C. The LINC00893 gene was cloned into the pEZ-Lv201 lentivirus vector (GeneCopoeia), RBFOX2 gene was cloned into the pcDNA3.1 vector (GeneCopoeia), and wild-type *TP53* were cloned into the pEnter lentivirus vector (Vigene Biosciences). All the small interfering RNAs (siRNA) and oligonucleotides (ASO) were purchased from RiboBio (China). All plasmids, siRNA and ASO were transfected with Lipofectamine 3000 reagent (Invitrogen) following the instructions. The sequences of siRNAs and ASO used are listed in Supplementary Table S5.

### RNA Sequencing and Bioinformatics Analysis

RNA sequencing was performed using an Illumina HiSeqTM 2500. Sample preparation was performed based on the manufacturer’s standard protocols. The cDNA/DNA/small RNA libraries were sequenced on the Illumina sequencing platform by Genedenovo Biotechnology Co., Ltd. (Guangzhou, China). The public datasets analyzed in the current study are available in TCGA (https://cancergenome.nih.gov/), GEPIA (http://gepia.cancer-pku.cn/), and KM Plotter (http://kmplot.com/analysis/). GSEA 3.0 (http://www.gsea-msigdb.org/gsea/index.jsp) was used to perform Gene set enrichment analysis (GSEA).

### LncRNA Coding Capacity Prediction

Strand-specific genomic coordinates for all exons of human LINC00893, PINCR, NEAT1, GAPDH, SDHA and UBC genes were downloaded from the UCSC Genome Browser (GRCh38/hg38) in BED format. PhyloCSF was applied to generate FASTA alignments to assess the coding potential (the codon substitution frequencies score - CSF) of individual exons and of mature transcripts of the above genes. The Coding Potential Assessment Tool (CAPT, http://lilab. research.bcm.edu/cpat/) constructs a logistic regression model built with four sequence features: open reading frame coverage, open reading frame size, hexamer usage bias and the Fickett TESTCODE statistic. The cutoff value for human coding probability (CP) was set at 0.364. CP < 0.364 was considered a noncoding transcript, whereas CP ≥ 0.364 was defined as a coding transcript.

### Real-Time Quantitative Reverse Transcription PCR (RT-qPCR)

RNA isolation plus (TaKaRa, Japan) was used to extract total RNA from tumor tissues or cells. Single-stranded cDNA was generated from 1 μg of total RNA in a 20 μL reaction volume system using oligodT primers according to the protocol supplied with Primer ScriptTM RT Reagent (TaKaRa, Japan). The relative expression levels were measured by qPCR using an ABI 7900HT instrument (Applied Biosystems, United States) in a total volume of 10 µL with the SYBR green detection system (Takara, Japan), and GAPDH was used as an endogenous control. The primers used for the qPCR analysis are listed in Supplementary Table S5.

### Proliferation Assay

Cell viability was examined by Cell Counting Kit-8 (CCK-8; Dojindo Laboratories, Japan). Briefly, after treatments, CCK-8 solution (10 μL) was added to each well, and the plates were incubated for an additional 2 h. The absorbance of each well was measured at 450 nm using a microplate reader (MultiskanEX, Lab systems, Finland). In the colony formation assay, treated cells (1×10^3^ cells/well) were seeded into 6-well plates and cultured in DMEM/F12. Then the medium was replaced every 3 days. After 14 days, colonies were fixed with paraformaldehyde and stained with 0.1% crystal. After that, colonies were counted.

### Transwell Assays

Firstly, GC cells were transfected as described above for 48 h. Next, cells were harvested and counted. Then a certain number of transfected cells (5×10^4^ cells/well) were cultured with serum-free medium in the upper chamber of Transwell plates (Corning, United States). Medium containing fifteen percent FBS was added into the lower chamber of the Transwell plates. For the invasion experiments, the upper chamber was covered with DMEM (F12) and Matrigel (BD Biosciences, CA) mixture in advance. Finally, cells on the top of the chamber were wiped with cotton swabs, while cells that went through the membrane were stained with 0.1% crystal violet, observed and counted under microscope at ×100 magnification.

### *In vivo* Assays

Four-week-old female athymic BALB/c nude mice were fostered under specific pathogen-free conditions and treated according to protocols. Lv201-LINC00893 or control vector were stably transfected into MKN45 cells. To assess the cell proliferation viability in different groups *in vivo*, the stable overexpressing cells were harvested, washed with cold PBS and resuspended to 5 × 10^7^ cells/ml. Then, 100 μL cell suspension was subcutaneously injected into the armpits of each mouse. Tumor growth was examined and recorded every week, and tumor volumes were calculated using equation V = 0.5 × D × d^2^ (V, volume; D, longitudinal diameter; d, latitudinal diameter). Four weeks after injection, mice were sacrificed, and two groups of subcutaneous tumors were obtained and imaged separately. In the cell metastasis assay *in vivo*, the transfected cells were resuspended at a concentration of 1 × 10^7^ cells/ml, and then 100 μL of resuspended cells was injected into the tail veins of the two groups of mice. Eight weeks after injection, the mice were euthanized. Lungs of the mice in two groups were removed and photographed. We then performed hematoxylin and eosin (HE) staining after tissue fixation and sectioning to evaluate the metastatic nodes, besides, the number of metastatic nodes in each lung was counted. The animal experiments were conducted in accordance with the Guide for the Care and Use of Laboratory Animals of the National Institutes of Health. Our whole protocol was approved by the Committee on the Ethics of Animal Experiments of the First Affiliated Hospital of Sun Yat-sen University.

### Nuclear and Cytoplasmic Extraction

Cytoplasmic and nuclear fractions of RNA were isolated in accordance with the instructions of PARIS™ Kit (AM1556, Thermo Fisher Scientific, United States). Briefly, AGS and MKN28 cells were washed with cold PBS and lysed in Cell Fraction Buffer on ice for 10 min. Then gathering the fractions into new tubes and centrifugating at 500 × g for 3 min at 4°C. After that, the supernatant was collected as the cytoplasmic fraction. Finally, washing the rest pellet with Cell Fraction Buffer and collecting the nuclei.

### Chromatin Isolation by RNA Purification (CHIRP)

RNA was extracted from AGS cells or transcribed *in vitro* and purified. Purified RNA was then incubated with specific prewashed beads at 37°C for 30 min. After centrifugation, the supernatant was hybridized with denatured biotin-labeled RNA probes at 55°C for 2 h. Next, we added prewashed beads, incubated them at 37°C for 1 h, centrifuged them, and washed the beads. The pulldown RNA was extracted and analyzed by quantitative reverse transcription PCR (RT-qPCR). LINC00893 RNAs were purified, and equal amounts of transcripts were used in the binding assays. The binding components were pulled down by biotin-labeled RNA probes and analyzed by western blotting.

### RNA Immunoprecipitation (RIP)

RNA immunoprecipitation (RIP) assays were performed using a Magna RIP RNA-Binding Protein Immunoprecipitation Kit (Millipore, United States) according to its instructions. Anti-RBFOX2 (Bethyl, United States) and normal Rabbit IgG (CST, United States) were used to immunoprecipitated with target RNA or as a negative control. Finally, RT-qPCR analysis was performed to detect the enrichment of the coprecipitated RNA binding to RBFOX2.

### Fluorescence *in situ* Hybridization (FISH)

Cy3 labeled LINC00893 probe was designed and synthesized by GenePharma (Shanghai, China). For FISH assay, AGS or MKN28 cells (1 × 10^5^) were fixed in 4% formaldehyde and permeabilized with 0.3% Triton X-100 for 15 min, washed with PBS three times and once in 2× SSC buffer. Hybridization was carried out using DNA probe sets at 37 °C for 16 h. Images were obtained with confocal laser scanning microscope (Zeiss, Germany) and processed using the ZEN imaging software.

### Western Blotting Analysis

Total proteins were extracted using RIPA supplemented with protease and phosphatase inhibitor reagents (Thermo-Fisher Scientific, United States). Equal amounts of protein samples were separated by SDS/PAGE and then electrophoretically transferred to PVDF membranes (Millipore, United States). Next, the membranes were probed with specific primary antibodies against p53 (dilution, 1:1,000; Abcam, United States), p21 (dilution, 1:1,000; CST, United States), RBFOX2 (dilution, 1:1,000; Bethyl, United States), E-cadherin (dilution, 1:1,000; CST, United States), N-cadherin (dilution, 1:1,000; CST, United States), vimentin (dilution, 1:1,000; CST, United States) and GAPDH (dilution, 1:5,000; Proteintech, China) at 4°C overnight, followed by incubation with HRP-conjugated secondary antibodies (dilution, 1:5,000; CST, United States) at room temperature for 1 h. Immunoreactive bands were visualized using enhanced chemiluminescence reagents (Bio-Rad, United States), and GAPDH was considered an inner loading control.

### Statistical Analysis

All data are presented as the mean ± standard deviation, appropriate statistical methods including Student’s t-test, Wilcoxon signed-rank test, Mann-Whitney test, Pearson chi-square test were used to calculate differences between groups, and *p* < 0.05 indicates statistical significance. Statistical analyses were performed using SPSS Statistics software (version 18.0) or GraphPad Prism 7 software.

## Results

### Decreased LINC00893 Predicts Poorer Survival in GC Patients

**FIGURE 1:**
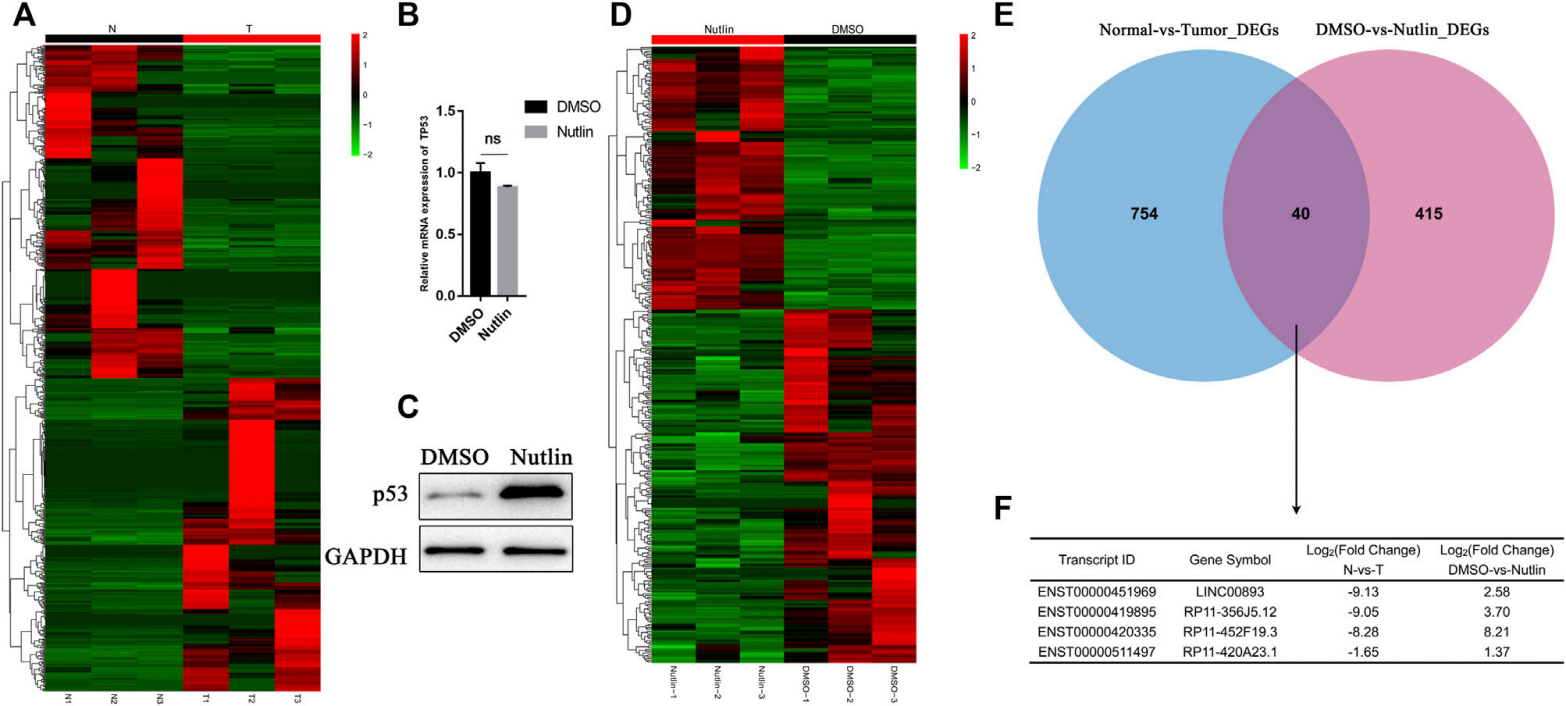
Identification of p53-regulated tumor suppressor lncRNAs in gastric cancer **(A)** Heat map is shown for the differentially expressed lncRNAs identified by RNA sequencing between three gastric cancer tissues and paired normal tissues **(B**,**C)** Isogenic *TP53*-WT AGS cells were untreated or treated with Nutlin-3 for 12 h, RT-qPCR and immunoblotting was performed for p53 and the loading control GAPDH **(D)** Heat map is shown for the differentially expressed lncRNAs identified by RNA sequencing in duplicate from AGS cells untreated or treated with nutlin-3 for 12 h. Upregulated genes are shown in red and downregulated genes in green **(E**,**F)** Venn diagram showing the overlap between the transcriptomes downregulated in gastric cancer tissues and the transcriptomes upregulated after nutlin-3 treatment of AGS cells.

**FIGURE 2:**
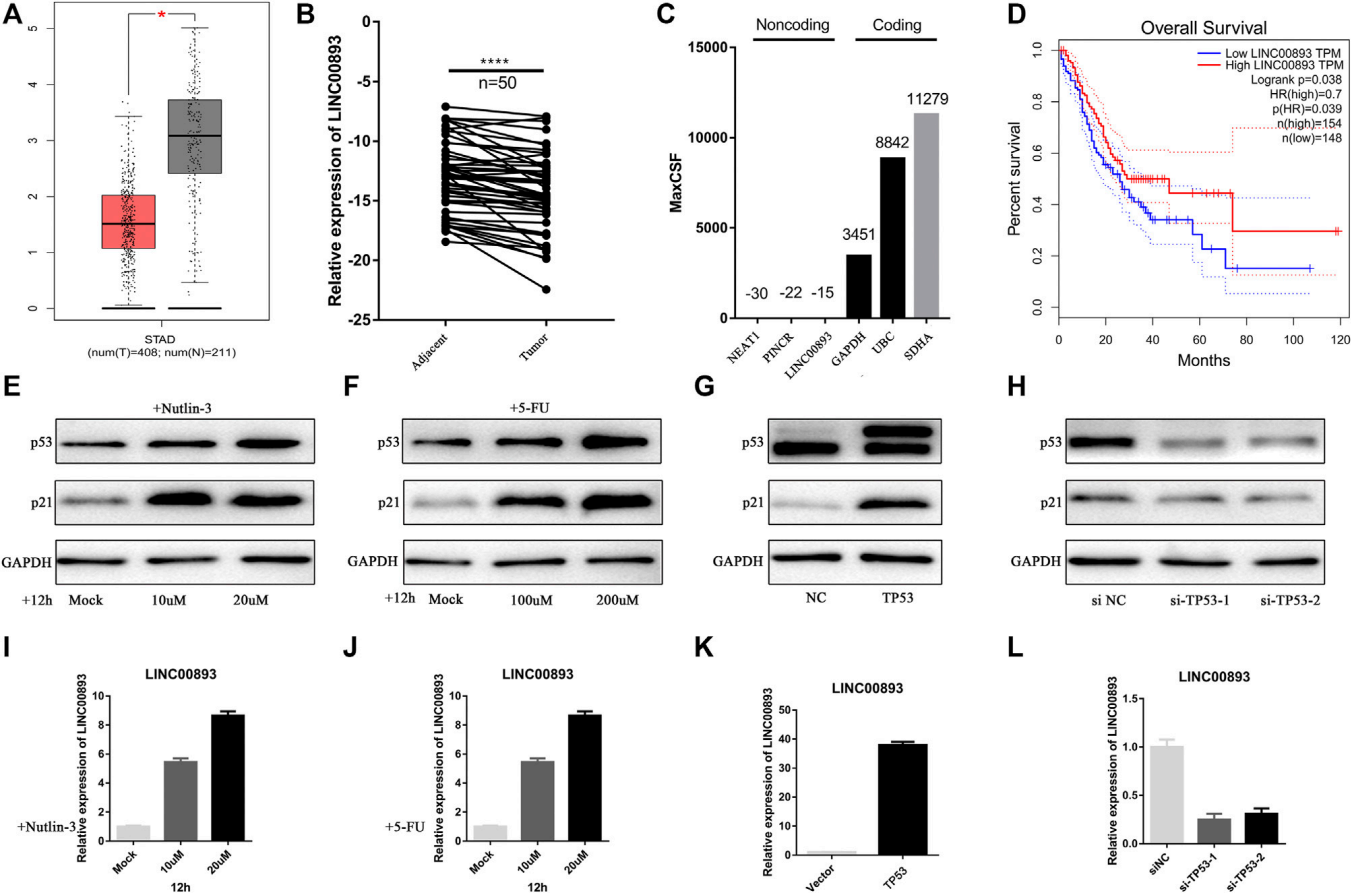
LINC00893 is significantly low-expressed in gastric cancer and regulated by p53 **(A)** Analysis of LINC00893 expression in unpaired GC (T = 408) and normal tissues (N = 211) in the GEPIA (*p* < 0.05) **(B)** Relative LINC00893 expression in 50 paired fresh frozen normal gastric tissues and gastric cancer tissues **(C)** Maximum CSF scores of LINC00893 as well as other coding and noncoding RNAs determined by analysis with PhyloCSF **(D)** Kaplan-Meier survival curve indicated the lower expression of LINC00893 associated with poorer survival rates **(E,F)** p53 and p21 expression in AGS treated with gradient nutlin-3 or 5-FU **(G,H)** p53 and p21 expression in AGS after transfection of *TP53* siRNA or overexpression plasmid **(I,J)** LINC00893 expression in AGS treated with gradient nutlin-3 or 5-FU **(K,L)** LINC00893 expression in AGS after transfection of TP53 siRNA or overexpression plasmid. *p < 0.05, ****p < 0.0001, all RT-qPCR results were normalized to GAPDH.

First, to screen out the potential tumor suppressor lncRNAs in GC, we performed RNA sequencing of three pairs of human GC tissues and matched nontumor tissues. Differentially expressed lncRNA transcripts were identified. A total of 409 lncRNA transcripts were found to have significant decreases in GC tissues vs nontumor tissues (Fold change<0.5, *p* < 0.05), while 385 were found to have significant increases. (Fold change>2, *p* < 0.05) (Figure 1A; Supplementary Table S1). Nutlin-3 is an inhibitor of human homolog of murine double minute 2 (MDM2), which is a negative regulator of p53 (Endo et al., 2011). AGS is a *TP53*-wild type GC cell line, in which nutlin-3 has been proved to significantly elevate the expression of p53 (Wei et al., 2010; Vaseva et al., 2011). To determine the optimal concentration and time of nutlin-3 stimulation, we treated AGS cells with gradient concentrations and time periods of nutlin-3 and then detected the expression of p53 and its downstream effector p21 (Supplementary Figure S1A–D). Afterwards, we treated AGS cells with nutlin-3 at the most appropriate concentration (10 µM) and time (12 h) and then conducted RNA sequencing to identify the differentially expressed lncRNA transcripts (Figures 1B,C). As the cluster map shows, 152 transcripts were downregulated when AGS was stimulated with nutlin-3 (Fold change<0.5, *p* < 0.05), while 146 transcripts were upregulated, which were considered potential targets of p53 (Fold change>2, *p* < 0.05) (Figure 1D; Supplementary Table S2). Next, we cross-analyzed the lncRNAs differentially expressed in gastric tissues and AGS cells elevated after the accumulation of p53, and we found there were 40 lncRNAs differentially expressed in both sequencing data (Figure 1E). Intriguingly, there were only four lncRNAs decreased in GC tissues (Fold change<0.5, *p* < 0.05) and, at the same time, upregulated in AGS cells treated with nutlin-3 (Fold change>2, *p* < 0.05) (Figure 1F). This might be due to the highly heterogeneous nature of GC tissues and huge differences of the lncRNA expression profiles between GC tissues and AGS cells. We used GEPIA (Tang et al., 2017) to analyze the expression of these four candidate lncRNAs (Figure 2A, Supplementary Figure S1E). In addition, RT-qPCR confirmed that the level of LINC00893 was significantly decreased in fresh gastric tumor tissues compared with nontumor tissues, while there were no differences in the other three lncRNAs (Figure 2B, Supplementary Figure S1F). We also found there were no significant differences between the expression of these three lncRNAs and the survival of GC patients (Supplementary Figure S1G). Therefore, we conducted further research on LINC00893. The noncoding capability of LINC00893 was confirmed by analysis with PhyloCSF (Lin et al., 2011) and the Coding Potential Assessing Tool (CPAT) (Duan et al., 2021) (Figure 2C, Supplementary Figure S1H).

In addition, the correlation of the expression of LINC00893 and the clinicopathological characteristics of patients was analyzed. As shown in Table 1, LINC00893 expression was negatively associated with tumor size, primary tumor (T) stage, lymph node metastasis, distant metastasis and tumor-node-metastasis (TNM) stage. Further analysis indicated that low expression of LINC00893 was negatively correlated with worse survival of GC patients (Figure 2D), suggesting that LINC00893 may inhibit GC progression.

**TABLE 1:** Correlation between LINC00893 expression and clinicopathological characteristics in GC patients.

Parameters	Cases number	LINC00893 expression		*P-* value
		Low	High	
Gender				0.769
Male	32	17	15	
Female	18	8	10	—
Age	—	—	—	0.742
<50	12	5	7	—
≥50	38	20	18	—
Tumor size	—	—	—	0.024
≤4 cm	23	5	18	—
>4 cm	27	10	7	—
Primary tumor (T) stage	—	—	—	0.042
T1-T2	20	6	14	—
T3-T4	30	19	11	—
Lymph node metastasis	—	—	—	0.027
Yes	24	17	7	—
No	26	10	16	—
Distant metastasis	—	—	—	0.010
Yes	26	18	8	—
No	24	7	17	—
TNM stage	—	—	—	0.024
I/II	27	11	16	—
III/IV	23	17	6	—

### LINC00893 Is Regulated by p53 Indirectly in GC Cells

To delineate the possible link to p53, we looked for a correlation between LINC00893 and p53. First, we found that nutlin-3 treatment significantly increased the expression of LINC00893 in AGS cells, which express wild-type (WT) p53 (Figures 2E,I). Next, we treated AGS cells with the chemotherapy drug 5-fluorouracil (5-FU), which could cause DNA damage and increase the level of p53. We found that LINC00893 increased with higher concentration gradients, consistent with p53 (Figures 2F,J). To rule out other effects of nutlin-3 and 5-FU, we transfected *TP53* plasmids or siRNAs and verified the expression of p53 (Figures 2G,H). With the overexpression of p53, LINC00893 was significantly elevated, while it was reduced after the knockdown of p53 (Figures 2K,L). Despite its p53-dependent induction, we were unable to identify an obvious p53 binding site in the vicinity of the LINC00893 promoter, nor was one identified in a recent p53 ChIP-seq study performed in *TP53* WT cells (Sanchez et al., 2014), thus suggesting that p53 may regulate LINC00893 expression via an indirect pathway, possibly through other p53 targets associated with transcription or p53 binding to enhancer regions. To identify promoter elements involved in p53-mediated regulation of LINC00893, we then analyzed the promoter of LINC00893 and predicted the potential transcription factors (TFs). Among the predicted TFs, Sp1 transcription factor (SP1) was predicted both in GeneCards and PROMO (Farre et al., 2003). Intriguingly, SP1 has been proved to be regulated by p53 and act as an intermediary TF between p53 and its target gene (Innocente and Lee, 2005; Li et al., 2014a). Therefore, we speculated that p53 regulates LINC00893 expression through p53/SP1/LINC00893 axis.

### LINC00893 Inhibits GC Proliferation, Migration and Invasion *in vitro*

**FIGURE 3:**
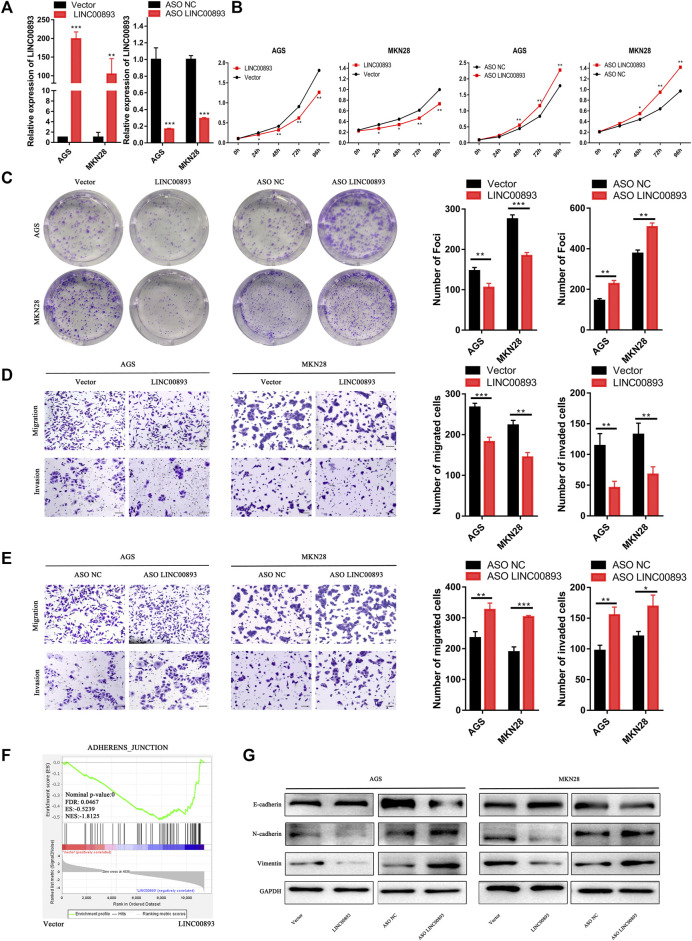
LINC00893 inhibits GC cell proliferation, migration, invasion and EMT *in vitro* **(A)** LINC00893 was overexpressed or knocked down in AGS and MKN28 cells **(B,C)** The effects of LINC00893 overexpression or knockdown on proliferation and plate colony-forming ability were measured in GC cells **(D,E)** The effects of LINC00893 overexpression or knockdown on migration and invasion were detected in GC cells **(F)** RNA-seq and Gene Set Enrichment Analysis (GSEA) of AGS treated with vector or LINC00893 overexpressed plasmid **(G)** Relative expression of E-cadherin, N-cadherin and Vimentin in GC cells after LINC00893 were either overexpressed or knocked down. *p < 0.05, **p < 0.01, ***p < 0.001.

According to the correlation between LINC00893 expression and GC, we focused on the biological functions of LINC00893 in GC proliferation and metastasis. AGS is an epithelial cell line derived from gastric adenocarcinoma and MKN28 is a highly metastatic GC cell line, which are widely used as models to detect the roles of oncogene or tumor suppressor gene in GC *in vitro*. The expression vector Lv201-LINC00893 or ASO-LINC00893 was transfected into GC cells, and the efficiency of LINC00893 overexpression or knockdown was confirmed by RT-qPCR (Figure 3A). CCK8 and colony formation assays were conducted, and they showed strikingly inhibited proliferation ability when LINC00893 was overexpressed or a promoting proliferation ability when LINC00893 was knocked down (Figures 3B,C). Transwell assays showed that LINC00893 overexpression significantly decreased the migration and invasion potential of GC cells, while LINC00893 knockdown showed the opposite effects (Figures 3D,E). To explore the downstream signaling pathways involved in LINC00893, we performed RNA-seq and bioinformatic analysis in AGS cells treated with empty vector or LINC00893 overexpression vector. Kyoto Encyclopedia of Genes and Genomes (KEGG) enrichment and Gene Set Enrichment Analysis (GSEA) results indicated that LINC00893 was correlated with pathways directly involved in the regulation of cell adherens junctions (Supplementary Table S3; Figure 3F; Supplementary Figure S1I). Therefore, we detected the key representative proteins of EMT. We found that overexpression of LINC00893 increased the level of E-cadherin and decreased the levels of N-cadherin and vimentin. The opposite effect was confirmed after knockdown of LINC00893 (Figure 3G). This suggests that LINC00893 can inhibit the process of EMT in GC cells.

### LINC00893 Suppresses GC Progression *in vivo*

**FIGURE 4:**
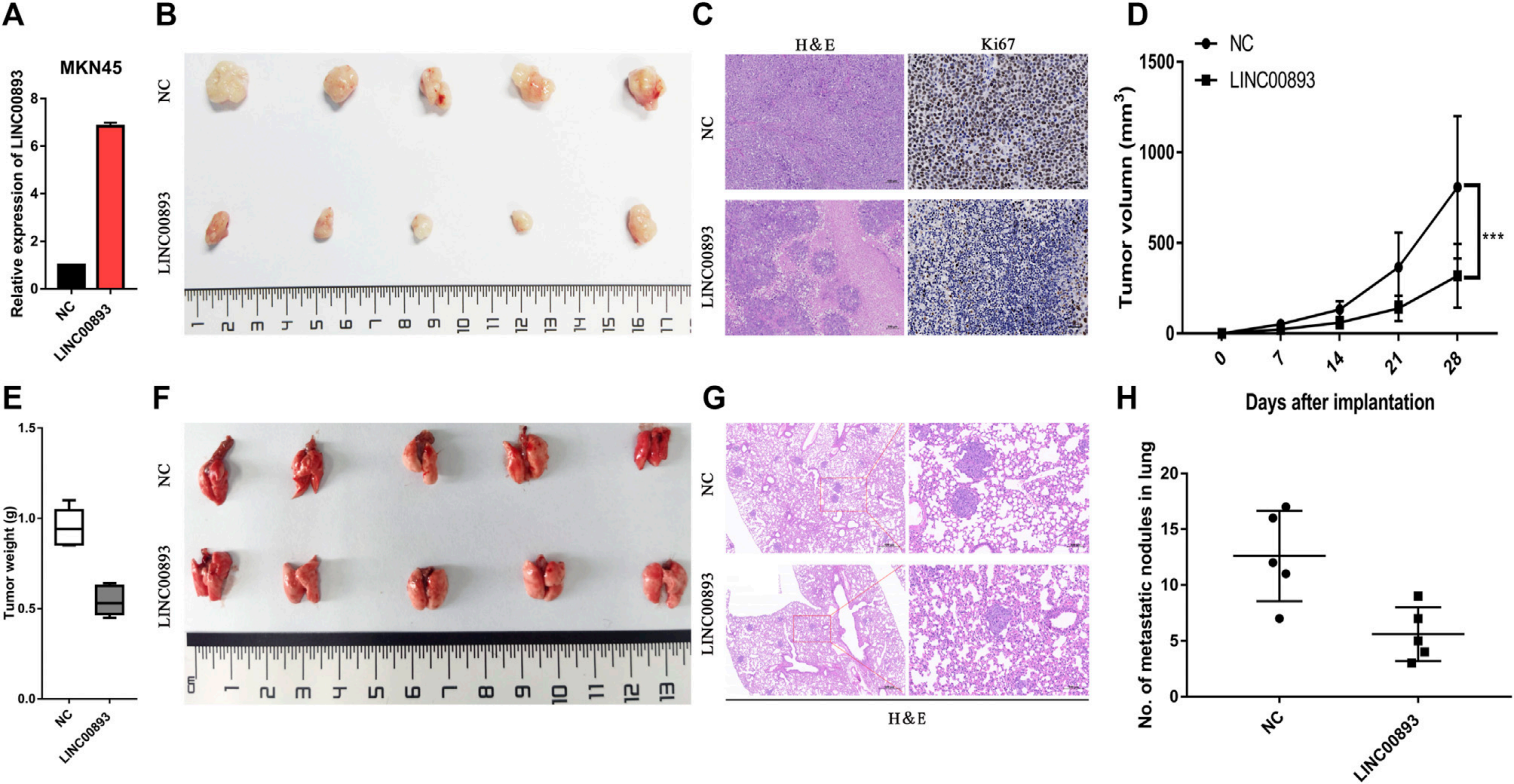
LINC00893 inhibits the growth and metastasis of gastric cancer cells *in vivo* **(A)** LINC00893 stable-overexpressed MKN45 cells was constructed **(B)** Representative images of subcutaneous xenograft tumors from the two group **(C)** Representative images of H&E and IHC with anti-Ki67 of the subcutaneous xenograft tumors **(D)** Quantification of tumor volumes in NC or LINC00893 overexpressed MKN45 cells in xenograft mouse models **(E)** Tumor weights were analyzed **(F)** Representative images of lung metastasis of LINC00893 overexpression groups and control group **(G)** Representative hematoxylin and eosin (H&E) staining results of corresponding lung metastatic nodules **(H)** Statistical analysis of numbers of metastatic nodules in the lung. ***p < 0.001.

To determine the roles of LINC00893 in GC tumorigenesis *in vivo*, MKN45, a GC cell line derived from gastric lymph nodes in a female patient with signet ring cell carcinoma, was used to construct model cells stably overexpressing LINC00893 or the control vector. Then, cells were subcutaneously injected into nude mice (Figure 4A). We found mice in the LINC00893-overexpressing group generated smaller tumors than the control group (Figure 4B), and HE staining was performed to identify the tumors (Figure 4C left panel). Tumor volume and weight also indicated that the overexpression of LINC00893 suppressed tumor growth *in vivo* (Figures 4D,E). Besides, a significant difference in Ki-67 staining was found between the LINC00893 and control group (Figure 4C right panel). Furthermore, by injecting the stably transfected MKN45 cells into tail veins of nude mice, it was found that LINC00893 dramatically inhibited the development of pulmonary metastasis (Figures 4F–H).

### LINC00893 Directly Binds to RBFOX2 and Decreases Its Expression

**FIGURE 5:**
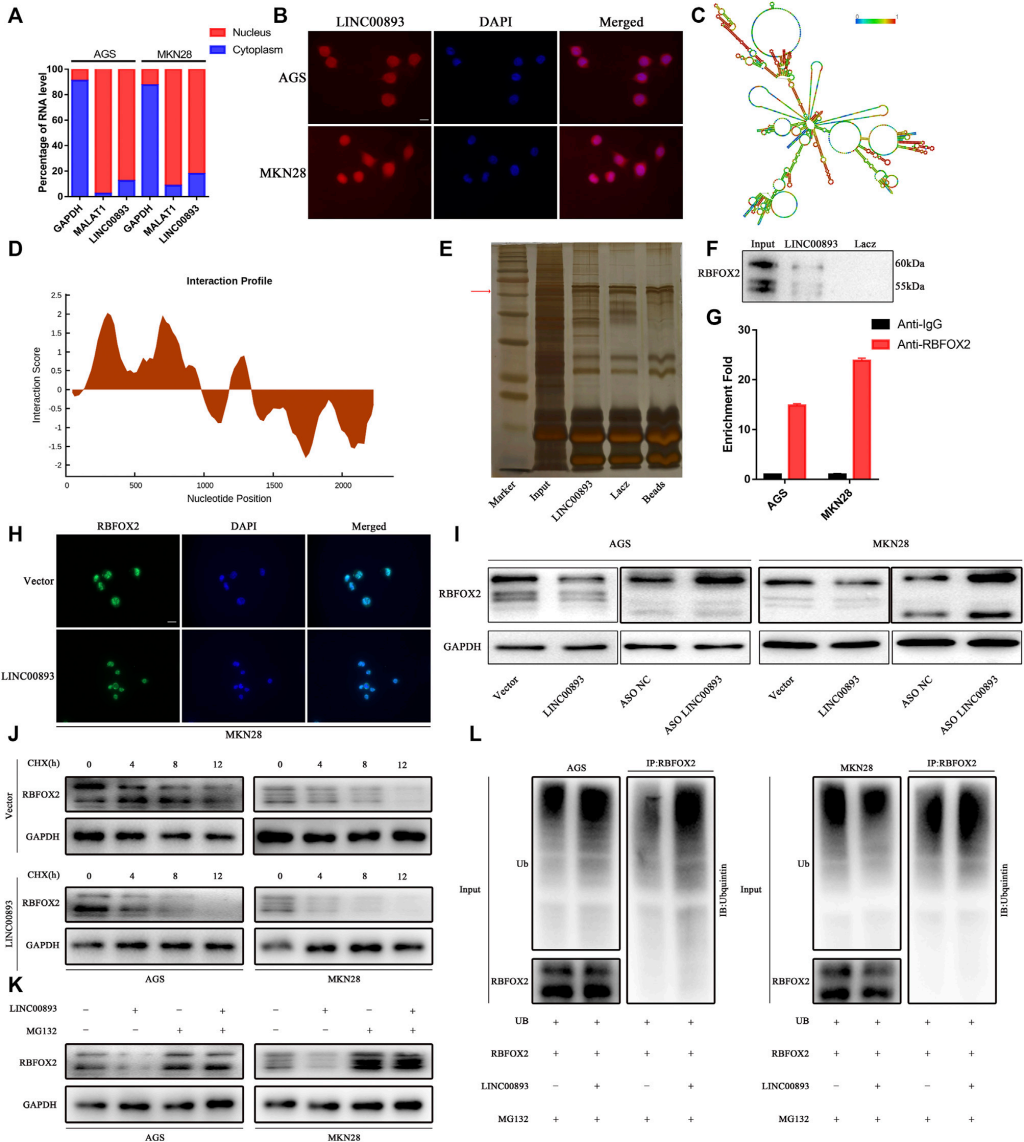
Identification of RBFOX2 as a binding partner for LINC00893 **(A)** RT-qPCR analysis from nuclear and cytoplasmic fractions of GC cells; the cytoplasmic GAPDH mRNA and the nuclear lncRNA MALAT1 were used as controls **(B)** Subcellular localization of LINC00893 detected by FISH. Scale bar: 20 μm **(C)** The secondary structure of LINC00893 was predicted (http://rna.tbi.univie.ac.at). The red color indicates strong confidence for the prediction of each base **(D)** The potential binding areas between LINC00893 and RBFOX2 were predicted using the catRAPID database **(E)** SDS-PAGE of proteins purified from CHIRP assay using biotinylated LINC00893 or antisense RNA **(F)** Western blotting analyses following CHIRP assays in AGS cells confirmed the interaction between LINC00893 and RBFOX2 **(G)** RIP assay followed by RT-qPCR suggested LINC00893 binds to RBFOX2 **(H)** Representative images of immunofluorescence staining for RBFOX2 expression in MKN28 cells after transfection with LINC00893 overexpression or empty vectors. Scale bar: 20 μm **(I)** Western blotting to analyze total level of RBFOX2 after LINC00893 were overexpressed or knocked down in GC cells **(J)** A CHX treatment was administered to assess RBFOX2 degradation in AGS and MKN28 cells **(K)** MG-132 abolished the downregulation of RBFOX2 protein expression induced by LINC00893 overexpression in AGS and MKN28 cells **(L)** Ubiquitination assays revealed that LINC00893 overexpression increased the level of the ubiquitinated RBFOX2 protein in AGS and MKN28 cells.

Several recent studies have revealed the closely related regulatory network between lncRNAs and proteins. GO (Gene Ontology) enrichment indicated that LINC00893 might play a role though binding to proteins (Supplementary Figure S2B). First, to identify the subcellular localization of LINC00893, we detected LINC00893 expression in cytoplasmic and nuclear fractions by RT-qPCR analysis (Figure 5A). The results showed that LINC00893 was localized predominantly in the nucleus, with some localization in the cytoplasm, which was further verified by RNA fluorescence *in situ* hybridization assays (Figure 5B). The secondary structure of LINC00893 was also predicted (Figure 5C). By using the StarBase database (Li et al., 2014), a large number of proteins were predicted to directly bind to LINC00893 (Supplementary Table S4). The splicing factor RNA Binding Protein Fox-1, Homolog 2 (RBFOX2) was included, which is a classic nuclear regulator of Polycomb Repressive Complex 2 (PRC2) recruitment to active genes, particularly those with bivalent histone modifications, to mediate dynamic transcriptional control and regulate autoregulation in RNA-binding protein networks (Venables et al., 2013; Braeutigam et al., 2014; Jangi et al., 2014; Wei et al., 2016). We found that nucleotides 753-848 of the LINC00893 sequence and amino acids 126-177 of the RBFOX2 sequence were the most likely binding areas (Figure 5D). To verify the direct interaction between LINC00893 and RBFOX2, western blotting was carried out using antibodies specific for RBFOX2 to test the protein samples precipitated by the LINC00893 CHIRP assay. This result indicated that RBFOX2 was pulled down by LINC00893 (Figures 5E,F). To further confirm this interaction, we carried out an RIP assay using a RBFOX2-specific antibody, followed by RT-qPCR using primers specific for LINC00893. As expected, LINC00893 was enriched in the anti-RBFOX2 group compared to the control IgG group (Figure 5G). These results indicated that RBFOX2 directly interacts with LINC00893.

Then, we examined RBFOX2 expression when LINC00893 levels were altered to determine whether LINC00893 regulated RBFOX2 expression. Interestingly, LINC00893 overexpression reduced the levels of the RBFOX2 protein (Figures 5H,I). According to our findings above, we inferred that LINC00893 regulated RBFOX2 protein stability. To elucidate the underlying mechanism by which LINC00893 regulated the RBFOX2 protein, we performed cycloheximide chase assays and observed an obvious shorter half-life of RBFOX2 in LINC00893-overexpressing cells than in empty control cells (Figure 5J). The ubiquitin-proteasome degradation pathway has been reported to be a primary mechanism regulating protein levels in cells. The proteasome inhibitor MG132 antagonized the decrease in RBFOX2 protein levels caused by LINC00893, indicating that LINC00893 downregulated the expression of RBFOX2 by promoting its proteasomal degradation (Figure 5K). Subsequent ubiquitination assays revealed that LINC00893 overexpression increased the level of the ubiquitinated RBFOX2 protein in AGS and MKN28 cells (Figure 5L). Taken together, LINC00893 directly interacts with RBFOX2 and promotes its ubiquitin-mediated degradation in GC cells.

### RBFOX2 Is Overexpressed in GC Cells and Promotes Its Progression

**FIGURE 6:**
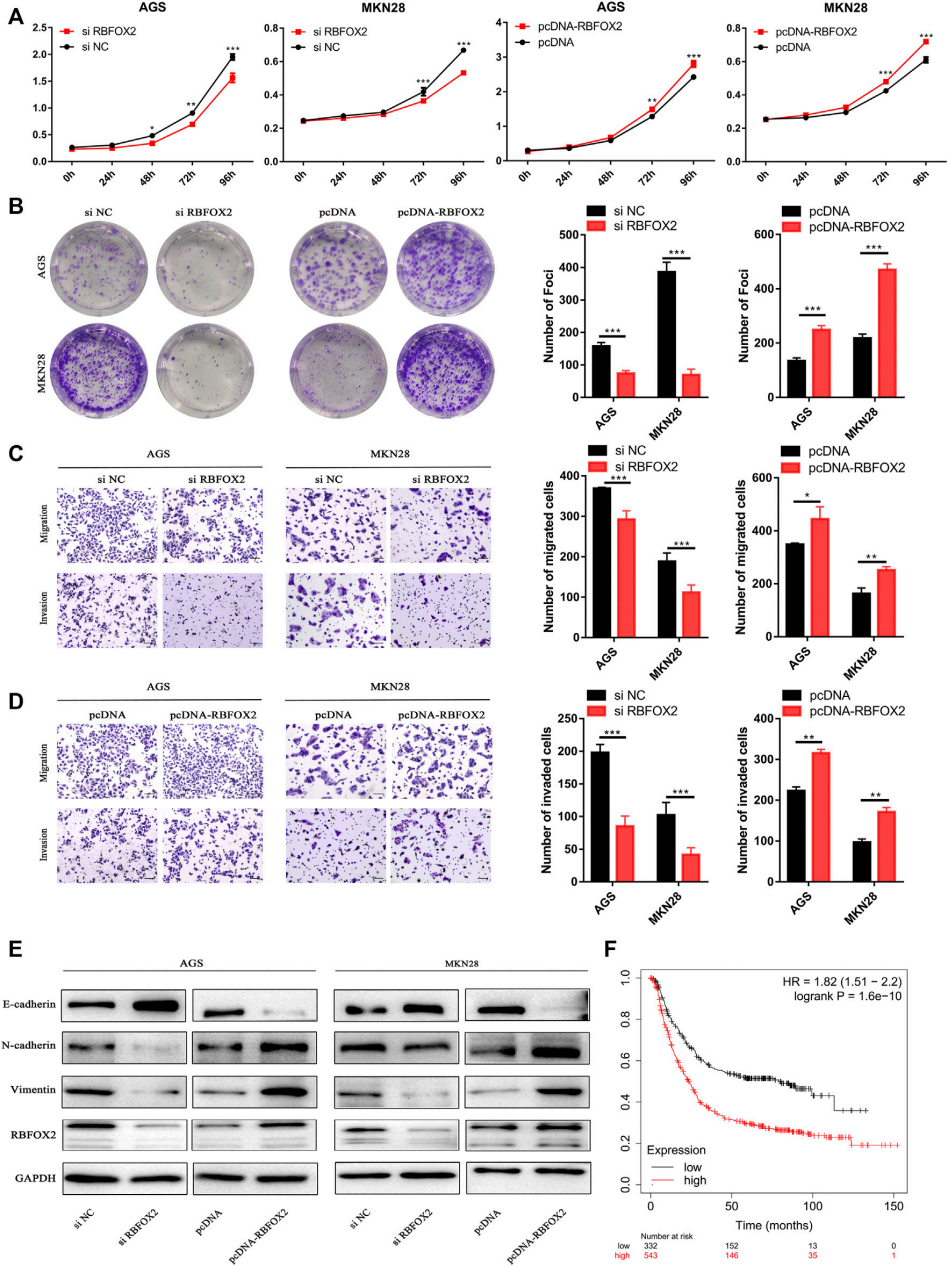
RBFOX2 promotes malignant behavior and EMT in GC cells *in vitro* **(A,B)** The effects of RBFOX2 knockdown or overexpression on proliferation and plate colony-forming ability were measured in GC cells **(C,D)** The effects of RBFOX2 knockdown or overexpression on migration and invasion were detected **(E)** Relative expression of E-cadherin, N-cadherin and Vimentin was detected by western blotting in gastric cancer cells after RBFOX2 were either knocked down or overexpressed **(F)** Survival rates analysis of between RBFOX2 and GC were analyzed by KM Plotter database (https://kmplot.com). *p < 0.05, **p < 0.01, ***p < 0.001.

RBFOX2 promotes tumor progression by acting as a splicer of EMT-related genes in several types of cancers (Shapiro et al., 2011; Braeutigam et al., 2014); however, the status and role of RBFOX2 in GC is unclear. Therefore, we focused on how RBFOX2 affects EMT-related genes and its role in GC cells. First, we knocked down RBFOX2 with siRNA or increased it by transfecting an overexpression plasmid. We found that the proliferation ability of GC cells was greatly decreased when RBFOX2 was downregulated but increased when RBFOX2 was upregulated (Figures 6A,B). The migration and invasion abilities of GC cells showed the same tendency (Figures 6C,D). Additionally, we found that when RBFOX2 was knocked down, the expression of E-cadherin increased, while N-cadherin and vimentin decreased, in accordance with the results when RBFOX2 was overexpressed (Figure 6E). In addition, analysis from KM Plotter (http://kmplot.com/analysis/) showed that high expression of RBFOX2 was correlated with a worse survival of GC patients, which is the opposite tendency with LINC00893 in terms of the overall survival rate (Figure 6F).

### The Effects of LINC00893 on GC Cells Are Mediated by RBFOX2

**FIGURE 7:**
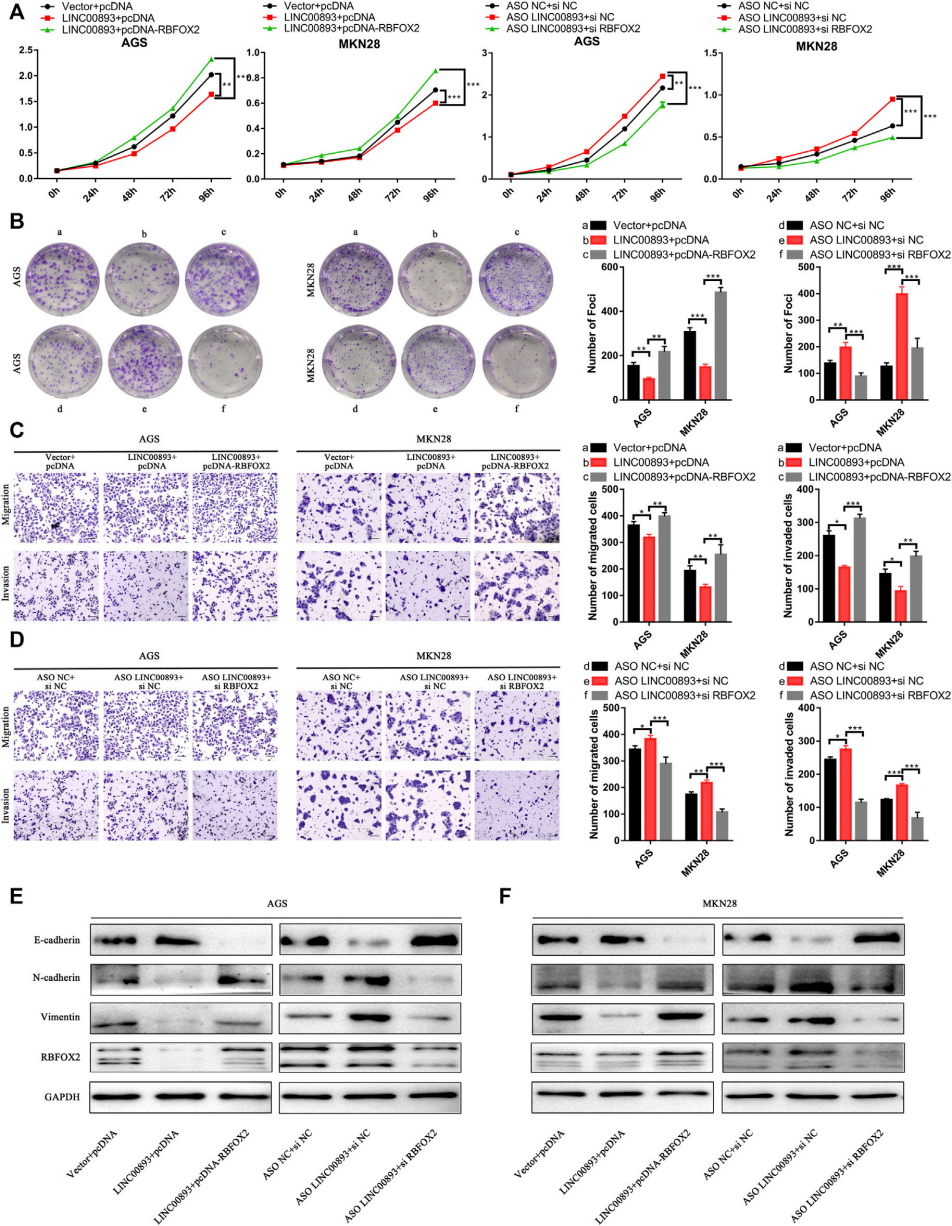
LINC00893 suppresses the proliferation and metastasis of GC cells through RBFOX2 **(A,B)** The effects of LINC00893 overexpression or knockdown on proliferation and plate colony-forming ability were rescued by RBFOX2 in GC cells **(C,D)** The effects of LINC00893 overexpression or knockdown on migration and invasion ability were rescued by RBFOX2 in GC cells **(E,F)** The effects of LINC00893 overexpression or knockdown on E-cadherin, N-cadherin and Vimentin were rescued by RBFOX2 in GC cells.

Subsequently, we determined whether LINC00893 exerts its function though RBFOX2. We found that the LINC00893-mediated suppression of proliferation, migration and invasion was remarkably abolished when we overexpressed LINC00893 and RBFOX2 in GC cells at the same time. Consistent with these results, transfecting siRNA of RBFOX2 and ASO of LINC00893 simultaneously strikingly eliminated the promotion of GC cell proliferation, migration and invasion induced by LINC00893 knockdown, suggesting that LINC00893-mediated RBFOX2 downregulation contributes to the suppression of LINC00893 on GC cell proliferation, migration and invasion (Figures 7A–D). In addition, the changes of the EMT-related proteins N-cadherin, vimentin and E-cadherin caused by the upregulation or downregulation of LINC00893 was rescued by the overexpression or knockdown of RBFOX2, respectively (Figure7E).

## Discussion

LncRNAs are classified as a large sort of transcripts with unknown biological roles and functions. Emerging researches have implied that dysregulation of certain lncRNAs could play a vital role in the initiation, tumorigenesis, and progression of GC (Tan et al., 2020; Xie et al., 2020; Yuan et al., 2020). LncRNA can crosstalk with chromatin, DNA, RNA, and proteins, regulating GC cell progression via transcriptional and posttranscriptional mechanisms (Kong et al., 2018; Zhuo et al., 2019). The cellular distribution of lncRNAs is informative for lncRNA functions (Shin et al., 2020). A large number of lncRNAs function by binding to a protein partner (Castello et al., 2016; Chen, 2016). Cytoplasmic lncRNAs mainly play roles in modulating mRNA stability or translation and thus influencing cellular signaling cascades, while nuclear lncRNAs are enriched for functionality involving chromatin interactions, transcriptional regulation, and RNA processing (Huang et al., 2018). A growing body of evidence has shown that lncRNAs can interact with proteins to modulate protein function, regulate protein-protein interactions, or directly localize within cellular compartments (Batista and Chang, 2013).

In this study, we analyzed tumor suppressor lncRNAs induced by p53 through RNA sequencing of GC tissues and p53-elevated AGS cells, and 40 lncRNAs were differentially expressed in both RNA sequence data. Some of these lncRNAs have been reported to play vital roles in GC and other cancers. LINC01140 promotes the progression and tumor immune escape in lung cancer (Xia et al., 2021). LncRNA GATA6-AS1 Inhibits LNM and EMT via FZD4 through the Wnt/β-Catenin Signaling Pathway in GC (Li et al., 2020). We screened out and explored an interesting lncRNA, LINC00893, which was downregulated in GC tissues but increased after the elevation of p53 in AGS cells. Moreover, LINC00893 regulated the proliferation, migration and invasion of GC cells *in vitro* and *in vivo*. Mechanistically, our results indicated that LINC00893 binds to RBFOX2 and regulates the subsequent ubiquitin-mediated degradation. Finally, we proved that LINC00893 suppresses the progression of GC cells through RBFOX2.

RBFOX2 is a regulator of alternative splicing involved in the EMT process and contributes to the increased invasiveness of cancer cells that have undergone EMT (Yeo et al., 2009). RBFOX2 has been linked to a proepithelial to EMT phenotype in several cancer types (Murphy et al., 2018; Choi et al., 2019). In the current study, we revealed the expression status and oncogenic role of RBFOX2 in GC cells *in vitro*. We also found that its expression can be regulated by LINC00893 through direct binding. KEGG and GO analyses showed that LINC00893 was related to ubiquitin-mediated proteolysis and ubiquitin-protein transferase activity. We verified that the RNA-protein binding of LINC00893 and RBFOX2 can influence the ubiquitination of RBFOX2 to reduce its expression, which is a mechanism often reported in the interactions of lncRNAs and proteins.

*TP53* is the most important tumor suppressor and the most frequently somatically mutated gene in human cancer; therefore, it is essential to figure out the functions of lncRNA components in the p53 network. Despite decades of studies on its significance in tumor suppression, the downstream effectors of the p53 response remain unclear. In recent years, increasing evidence has revealed that both proteins and noncoding genes including lncRNAs are involved in p53 regulatory networks. LncRNAs regulated by p53 in indirect ways play critical roles in genomic stability, DNA damage repair, cell cycle arrest, and apoptosis, which contributes to the tumor-suppressor functions of p53 (Chaudhary and Lal, 2017). Here, we discovered a lncRNA, LINC00893, regulated by p53 indirectly in GC cells, which enriched the p53 regulatory network, yet the specific mechanism by which p53 regulates LINC00893 remains to be verified. As we mentioned above, some other lncRNAs are also regulated by p53 in different ways. We infer that there may be connections between LINC00893 and them in the p53 regulatory network. It is possible that there is a synergic effect between LINC00893 and those regulated by p53 positively, such as lincRNA-p21, GUARDIN and TP53TG1 (Bao et al., 2015; Diaz-Lagares et al., 2016; Hu et al., 2018). On the other side, an antagonic effect may be possible between LINC00893 and those negatively regulated by the WT p53 pathway, such as LINP1(Zhang et al., 2016). However, further investigations are needed to verify these hypotheses.

In conclusion, we demonstrate that LINC00893 promotes the degradation of RBFOX2 by direct binding, thereby suppressing GC progression by decreasing the EMT and functional ability of GC cells. LINC00893 could serve as a prognostic indicator and potential therapeutic target for GC patients.

## Data Availability

The datasets presented in this study can be found in online repositories. The names of the repository/repositories and accession number(s) can be found below: Sequence Read Archive (SRA) of RNA-seq data, https://www.ncbi.nlm.nih.gov/sra. Accession numbers: PRJNA792194 and PRJNA792206.
