# Supplementary material for: p53-Induced LINC00893 Regulates RBFOX2 Stability to Suppress Gastric Cancer Progression
Source: Front Cell Dev Biol. 2022 Jan 19;9:796451. doi: 10.3389/fcell.2021.796451 (PMC8807521; doi:10.3389/fcell.2021.796451)
Supplement: Supplementary file 3 [file Table5.docx]

| **Table S5** | | |
| --- | --- | --- |
| **Sequences of primers, siRNAs and ASO** | | |
| **Primers Used for Quantitative Real-Time PCR** | | |
| **Gene** | | **Primer sequence** |
| *LINC00893* | |  |
| Forward | | CTGCACCTTCACTCTGGTCA |
| Reverse | | CCAATGACACCAGCCCTTAT |
| *GAPDH* | |  |
| Forward | | ACAACTTTGGTATCGTGGAAGG |
| Reverse | | GCCATCACGCCACAGTTTC |
| *TP53* | |  |
| Forward | | GTGACACGCTTCCCTGGATT |
| Reverse | | TGTTTCCTGACTCAGAGGGG |
| *MALAT1* | |  |
| Forward | | AATGTTAAGAGAAGCCCAGGG |
| Reverse | | AAGGTCAAGAGAAGTGTCAGC |
| **siRNA information** | | |
| **Target gene** | | **Sequence** |
| *TP53*-#1 | | GAGAAUAUUUCACCCUUAA |
| *TP53*-#2 | | UGGUUCACUGAAGACCCAGUU |
| *RBFOX2* | | GGGAUUCGGGUUCGUAACU |
| **ASO information** | | |
| **Target gene** | |  |
| *LINC00893* | | \| ATTACTGCGTGGTCACAAGG \| \| --- \| |
| **ChIRP probe set** | | |
| **Probe** | | **Sequence** |
| P1 | | CATCAGGCACTCCAGAGGCA |
| P2 | | GGAGTGAGAGTGAGGAGCAG |
| P3 | | CTGCAAAAGATGTAAGAACA |
| P4 | | TGTCCTCCCTCCCAGGAGTG |
| P5 | | GTGAACGCTCAGCCAAGGCA |
| P6 | | CAGAGAACAGACAGTGGTGA |
| P7 | | CCATCTGCTTTCTGACATAC |
| P8 | | GAGTGAAGGCAGATCAGACG |
| P9 | | CATCTTCTCAAATCCACCTG |
| P10 | | ACTGAGCAAGATGTCCTTGC |
| **LINC00893 RNA FISH probe set** | | |
| **Probe** | | **Sequence** |
| P1 | TCTCAC+TGAGGGAAGAAAGACA | |
| P2 | CAAAACAGCCC+TGCAAAAGATG | |
| P3 | ACAGACAG+TGG+TGAG+TATATTT | |
| P4 | GAAAACC+TGAGCTTGGAGGAAA | |
| P5 | AT+TCAAACCA+TCTTT+TGCAAGG | |
