# Supplementary material for: p53-Induced LINC00893 Regulates RBFOX2 Stability to Suppress Gastric Cancer Progression
Source: Front Cell Dev Biol. 2022 Jan 19;9:796451. doi: 10.3389/fcell.2021.796451 (PMC8807521; doi:10.3389/fcell.2021.796451)
Supplement: Supplementary file 6 [file DataSheet1.docx]

Supplementary Material

# Supplementary Figures and Tables

## Supplementary Tables

Table S1 Differentially expressed lncRNAs in the RNA-seq data of gastric cancer and adjacent tissues

Table S2 Differentially expressed lncRNAs in the RNA-seq data of AGS cells treated by nutlin-3a or DMSO.

Table S3 KEGG analysis of mRNA sequencing data of AGS cells transfected with empty control or LINC00893 plasmid.

Table S4 RNA-binding-proteins(RBP) of LINC00893 predicted by StarBase Database.

Table S5 Sequences of primers, siRNAs and ASO.

## Supplementary Figure


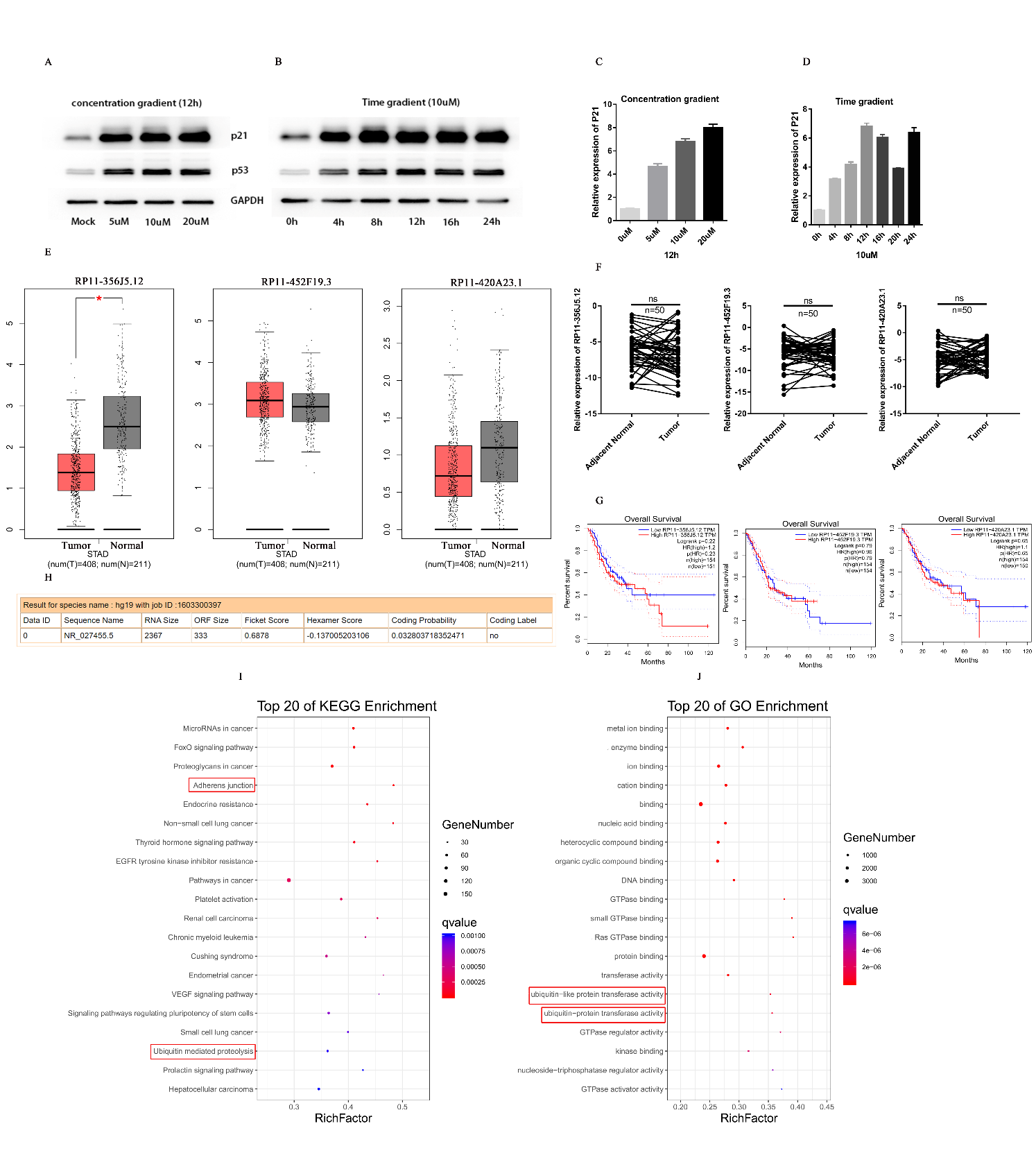


**Supplementary Figure 1.** (A-D): p53 and p21 expression in AGS cells treated with nutlin-3 of gradient time or concentration. (E): Analysis of RP11-356J5.12, RP11-452F19.3, RP11-420A21.3 expression in unpaired GC (T = 408) and normal tissues (N = 211) in the GEPIA. (F): The expression of the 3 candidate lncRNAs in GC tissues or adjacent normal tissues by RT-qPCR. (G): Kaplan-Meier survival curve of the 3 candidate lncRNAs from GEPIA. (H): Schematic of the coding potential of LINC00893 predicted by CPAT (http://lilab.research.bcm.edu/cpat/). (I), (J): KEGG and GO analysis of AGS treated with vector or LINC00893 overexpressed plasmid. *P < 0.05, ns: P>0.05.

## Raw Data

Raw data is available on the website https://www.jianguoyun.com/p/DfQPtPgQ6cb6CRiu3ZUE
